# Newborn clavicle fractures: 5 year-review of a tertiary-care hospital

**DOI:** 10.61622/rbgo/2025rbgo92

**Published:** 2025-11-18

**Authors:** Joana Rita Pinto Galvão, Mariana da Silva Leal, Margarida Neves Maciel da Silva, Nuno Miguel Sanches de Almeida, Carla Isabel Pereira Faustino Ferreira, Inês Maria Moreira Guedes Maia Nunes de Melo Seco

**Affiliations:** 1 Unidade Local de Saúde Gaia e Espinho Department of Obstetrics and Gynaecology Vila Nova de Gaia Portugal Department of Obstetrics and Gynaecology, Unidade Local de Saúde Gaia e Espinho, Vila Nova de Gaia, Portugal.; 2 Unidade Local de Saúde Gaia e Espinho Department of Pediatrics/Neonatology Vila Nova de Gaia Portugal Department of Pediatrics/Neonatology, Unidade Local de Saúde Gaia e Espinho, Vila Nova de Gaia, Portugal.; 3 University of Porto Center for Health Technology and Services Research Porto Portugal Center for Health Technology and Services Research, University of Porto, Porto, Portugal.; 4 University of Aveiro Department of Medical Sciences Aveiro Portugal Department of Medical Sciences, University of Aveiro, Aveiro, Portugal.

**Keywords:** Birth injuries, Clavicle/injuries, Fractures, bone, Infant, newborn

## Abstract

**Objectives:**

Clavicle fractures are the most common birth-related fractures. They may cause pain, arm mobility impairment, and brachial nerve injury (temporary or definitive). Therefore, we aimed to review the incidence, risk factors and prognosis of neonatal clavicle fractures in our hospital.

**Methods:**

All cases of clavicle fractures diagnosed and registered during the neonatal period between 2018 and 2022, in a tertiary-care Portuguese hospital, were reviewed. Statistical descriptive analysis, odds ratios (ORs), and 95% confidence intervals(95%CI) were calculated.

**Results:**

A total of 8132 births and 91 clavicle fractures were identified (1.1% incidence) – 0.04% (1/2512) in cesarean sections and 1.7% (90/5453) in vaginal births. An instrumental vaginal birth doubled the probability of clavicle fractures (OR 2.072, 95%CI 1.365-3.145 compared to spontaneous births). In 15.4% of clavicle fractures, births were complicated by shoulder dystocia. Shoulder dystocia significantly increased the risk of clavicle fracture (OR 35.71; 95% CI 17.86-71.43). Of the 91 cases, 22% resulted from pregnancies complicated by gestational diabetes; in these cases, the risk of clavicle fractures increased 76% (OR 1.761, 95%CI 1.068-2.904). Most of the cases were referred to a Neonatology follow-up appointment (92.3%). Fifteen neonates had a clinical suspicion of brachial plexus injury, but all of them regained normal arm mobility after physiotherapy.

**Conclusion:**

The incidence of clavicle fractures in our hospital is comparable to the literature (1.1%). To reduce the incidence of clavicle fractures not only its risk factors must be reduced, but a simulation-based labor ward team training program on shoulder dystocia should also be implemented.

## Introduction

Clavicle fractures are the most common fracture in neonates,^(1–4)^ with the incidence ranging from 0.05% to 3.5% in living births,^(2,5)^ but their incidence is often underestimated.^(1,5,6)^ It is much more common in vaginal deliveries than in cesarean sections.^(3,7)^ These fractures may cause pain, arm mobility impairment, and brachial nerve injury (temporary or definitive),^(4)^ and are responsible for parents’ anxiety and concern in the neonatal period.^(7,8)^ Displaced fractures are usually easily identified due to local deformity, crepitus, and neonate's discomfort aroused by passive mobility of the arm, but non-displaced fractures may only be diagnosed when bone callus formation is palpated.^(4)^

Several risk factors for neonatal clavicle fractures have been proposed, including birthweight,^(2–4,6)^ instrumental birth,^(3,4,6,9)^ shoulder dystocia,^(2,4,6,9,10)^ term birth^(1)^ and gestational diabetes.^(2,4,11)^ An isolated neonatal clavicle fracture also seems to increase the odds of shoulder dystocia at a subsequent delivery.^(12)^ Despite scientific controversy, a population-based study suggest that, in the setting of shoulder dystocia, clavicle fracture is not associated with a significant change in the risk of brachial plexus birth palsy.^(13)^

The treatment is usually conservative, involving gentle mobilization and pain management if needed.^(4)^ Eventually, neonatal fractures might also be the first sign of some metabolic or systemic disease.^(4)^ Nevertheless, the prognosis is overall good.^(3,4,6)^

In this paper, we aimed to review the incidence, epidemiology, risk factors and prognosis of neonatal clavicle fractures in our hospital.

## Methods

All neonates born in our hospital – a tertiary care Portuguese hospital - undergo a detailed physical examination, performed by a neonatologist, on the day of their clinical discharge. In this retrospective clinical study, all cases of diagnosed neonatal clavicle fractures (informatically codified by nationally certified doctors) between 2018 and 2022, in our center, were reviewed. For each case, the following data was collected: side of the clavicle fracture, gestational age at birth, type of birth (spontaneous vaginal birth, vacuum-assisted birth, forceps-assisted birth and cesarean section), healthcare professional responsible for the delivery (midwife, OBGYN medical resident, OBGYN specialist doctor), neonate birth weight, five minute APGAR score, previous maternal number of vaginal births, maternal body mass index at the onset of pregnancy, occurrence of shoulder dystocia (yes/no), confirmation of the fracture by radiography (yes/no), suspicion of brachial plexus lesion (yes/no); and if yes, whether the suspicion was temporary or permanent), follow-up in Neonatology clinic (yes/no) and need for physiotherapy (yes/no). The total number and type of deliveries, number of pregnancies complicated by gestational diabetes, births complicated by shoulder dystocia, and deliveries assisted only by nurse-midwives in each year of the study, were also registered. Data was adequately anonymized. Statistical descriptive analysis, odds ratios (ORs), and 95% confidence intervals (95% CI) calculations were performed using SPSS-26. This research did not receive any specific grant from funding agencies in the public, commercial, or not-for-profit sectors.

## Results

A total of 8132 births and 91 clavicle fractures were identified (1.1% incidence). A descriptive analysis of several variables concerning these births is available in table 1.

**Table 1:** Variables of the cases of clavicle fractures are descripted in detail

Variables	Results n(%)
Year of birth	2018 – 26(28.6)
	2019 – 16(17.6)
	2020 – 22(24.2)
	2021 – 11(12.1)
	2022 – 16(17.6)
Diabetes	Gestational Diabetes: 20(22)
	Preexisting Diabetes Mellitus: 0(0)
	No diabetes in pregnancy: 71(78)
Fractured clavicle	Left - 45(49.5)
	Right – 46(50.5)
Gestational week at birth	36 weeks – 3(3.3)
	37 weeks – 6(6.6)
	38 weeks – 12(13.2)
	39 weeks – 34(37.4)
	40 weeks – 28(30.8)
	41 weeks – 8(8.8)
Type of birth	Spontaneous vaginal birth – 46(50.5)
	vacuum-assisted vaginal birth – 43(47.3)
	Forceps-assisted vaginal birth – 1(1.1)
	Cesarean section – 1(1.1)
Birthweight (split by categories)	2000-2499g – 1(1.1)
	2500-2999g – 9(9.9)
	3000-3499g – 38(41.8)
	3500-3999g – 34(37.4)
	4000-4499g – 9(9.9)
Number of previous maternal vaginal births	Zero vaginal births – 45(49.5)
	One vaginal birth – 32(35.2)
	Two vaginal births – 9(9.9)
	Three vaginal births – 5(5.5)
Maternal body mass index at onset of pregnancy	Underweight (BMI <18.5) – 2(2.3)
	Normal weight (18.5≤ BMI ≤ 24.9) – 47 (53.4)
	Overweight (25< BMI < 29.9) – 17(19.3)
	Obesity (BMI ≥ 30) – 22(25)
	Missing – 3
Shoulder dystocia	Yes – 14(15.4)
	No – 77(84.6)
Clavicular fracture confirmed by radiography	Yes – 71(78)
	No – 20(22)
Temporary suspicion of brachial plexus injury	Yes – 15(16.5)
	No – 76(83.5)
Permanent suspicion of brachial plexus injury	No – 91(100)
Referral to post-discharge Neonatology clinic	Yes – 81(89)
	Yes, but missed it – 3(3.3)
	No – 7(7.7)
Need for physiotherapy	Yes – 14(15.4)
	No – 77(84.6)
Healthcare professional responsible for the birth	Nurse-Midwife - 34(37.4)
	OBGYN Medical Resident - 29(31.9)
	OBGYN Specialist Doctor – 28(30.8)

This incidence varied according to the mode of delivery: 0.04% (1/2512) in cesarean sections and 1.7% (90/5453) in vaginal births: 1.2% (46/3715) in spontaneous vaginal deliveries, 1.3% (1/75) in forceps-assisted and 2.6% (43/1663) in vacuum-assisted births. An instrumented vaginal birth doubled the probability of clavicle fractures, with an OR 2.072, 95%CI 1.365-3.145, compared to spontaneous vaginal births (OR 2.117, 95%CI 1.19-3.22 vacuum-assisted versus spontaneous vaginal births, OR 1.078, 95%CI 0.147-7.920 forceps-assisted versus spontaneous vaginal births).

Over the five years considered, the highest incidence was recorded in 2018 and the lowest in 2021 (1.5% vs 0.8%). The difference in incidence between the five years was non-significant (Chi-Square 5.852; p=0.211).

The majority (96.7%) of clavicle fractures occurred in term births, whereas the overall rate of prematurity in our hospital, during that time, was 10.6%. Only 9.9% of the newborns weighed 4000g or more.

Gestational diabetes was present in 22.0% of the pregnancies amongst the neonates with clavicle fractures. No case of preexisting diabetes was observed. Of the total of births, in 1129 (13.9%) gestational diabetes was diagnosed. Having gestational diabetes was significantly associated with 1.8 times the chance of clavicle fracture at birth (Yates's correction for continuity 4.382, p=0.036; OR 1.761, 95%CI 1.068-2.904).

Clavicle fractures occurred in 15.4% of births complicated by shoulder dystocia. In this period there were 42 cases of shoulder dystocia in the 5453 vaginal births (with 90 clavicle fractures) - Yates's correction for continuity 242.444, p<0.001. A neonate whose birth was complicated by shoulder dystocia was 36 times more likely to have a clavicle fracture (OR 35.71; 95% CI 17.86-71.43).

Around half (49.5%) of the clavicle fractures occurred in primiparas. A quarter of the pregnant women were obese (defined as body mass index over 30 at the onset of pregnancy), compared with the overall rate of 16.2% of obese pregnant women in this period – Yate's correction for continuity 4.505, p=0.034; OR 1,743, 95%CI 1.072-2.835.

Concerning fractures in spontaneous vaginal births, 26.1% (12/46) occurred in births assisted by doctors, and 73.9% (34/46) in births assisted only by nurse-midwives – a non-significant difference compared to the overall 24.6% spontaneous vaginal births assisted by doctors (OR 1.10, 95%CI 0.66-1.75).

Documentation by clavicle radiography was available in 78% of fractures. In the other cases, the diagnosis of clavicle fracture was only supported by clinical examination.

Most of the newborns (92.3%) were referred to a Neonatology follow-up appointment; all the seven newborns who were not referred to follow-up did not present arm movement impairment or other risk factors, concerning or not the clavicular fractures. Nevertheless, 3.3% (3/91) of the infants missed the planned follow-up appointment. Fifteen (16.5%) neonates had a suspicion of brachial plexus injury, but all of them regained normal arm mobility before being discharged (either from the neonatal stay or from the Neonatology clinic). From these 15 neonates, 13 had physiotherapy treatments and two had spontaneous resolution of the mobility impairment. Another neonate, despite no suspicion of brachial plexus injury, had a concomitant congenital muscular torticollis, and was thus referred to physiotherapy; therefore, a total of 14 infants underwent physiotherapy.

## Discussion

The limitations of the study include the potential imprecise records of diagnosis with a degree of subjectivity, such as shoulder dystocia. A multivariable logistic regression analysis was not feasible due to the lack of individual-level data for deliveries without clavicle fractures (only aggregated data were available). The strengths of this study are the high number of births under record, and the systematic specialized examination undergone by every neonate.

Several risk factors identified in our sample, including instrumental delivery, shoulder dystocia, and gestational diabetes, have been consistently associated in the literature with an increased risk of neonatal clavicle fracture. Instrumental vaginal delivery increases the likelihood of birth trauma due to the higher traction forces applied during delivery maneuvers.^(10)^ Gestational diabetes and shoulder dystocia are both associated with a greater difference between transverse abdominal and biparietal diameters,^(14)^ and this abdominal-to-head disproportion increases the risk of shoulder impaction. Furthermore, the maneuvers used in the resolution of shoulder dystocia have been shown to influence the number of clavicle fractures.^(15,16)^

To reduce the incidence of clavicle fractures, we believe its risk factors should be minimized: an instrumental birth should only be decided when a clear obstetric criterion^(17)^ is met; to minimize the number of shoulder dystocia, macrossomia and elevated birthweight should be prevented with appropriate maternal weight and weight gain control (often with a multidisciplinary approach), and eventual earlier induction of labor.^(16)^ Regular, multi-disciplinary, simulation-based and hands-on training in shoulder dystocia and other birth complications might be helpful.^(8,18–20)^ Nevertheless, a nationwide population-based Finnish study confirmed the decreasing number of neonatal clavicle fractures between 2004 and 2017,^(2)^ but, as many clavicle fractures happened in births with few to none risk factors, deemed the fundamental grounds for this decline as unclear.

To conclude, this study highlights the importance of risk factors such as instrumented birth, gestational diabetes and, more significantly, shoulder dystocia in neonatal clavicle fractures. With follow-up and physiotherapy, the prognosis is nevertheless good.

## Conclusion

The 1.1% incidence of clavicle fractures in our hospital is on the higher end of the incidence reported in the literature. An instrumental delivery was associated with a doubled risk of a clavicle fracture in vaginal births. Shoulder dystocia was associated with 36 times more risk of clavicle fractures. Gestational diabetes was common among these cases. These risk factors identified in our study are consistent with the literature. Most cases were followed-up in the Neonatology clinic; 16.5% had a suspicion of nerve injury, but no infant had a permanent lesion. To reduce the incidence of clavicle fractures, we believe, its risk factors should be reduced as possible, and a regular simulation-based labor ward multidisciplinary team training program on shoulder dystocia ought to be implemented.
